# Chloroplast Chaperonin: An Intricate Protein Folding Machine for Photosynthesis

**DOI:** 10.3389/fmolb.2017.00098

**Published:** 2018-01-19

**Authors:** Qian Zhao, Cuimin Liu

**Affiliations:** ^1^State Key Laboratory of Plant Cell and Chromosome Engineering, Institute of Genetics and Developmental Biology, Chinese Academy of Sciences, Beijing, China; ^2^University of Chinese Academy of Sciences, Beijing, China

**Keywords:** chaperonin, Rubisco, chloroplast, photosynthesis, protein folding

## Abstract

Group I chaperonins are large cylindrical-shaped nano-machines that function as a central hub in the protein quality control system in the bacterial cytosol, mitochondria and chloroplasts. In chloroplasts, proteins newly synthesized by chloroplast ribosomes, unfolded by diverse stresses, or translocated from the cytosol run the risk of aberrant folding and aggregation. The chloroplast chaperonin system assists these proteins in folding into their native states. A widely known protein folded by chloroplast chaperonin is the large subunit of ribulose 1,5-bisphosphate carboxylase/oxygenase (Rubisco), an enzyme responsible for the fixation of inorganic CO_2_ into organic carbohydrates during photosynthesis. Chloroplast chaperonin was initially identified as a Rubisco-binding protein. All photosynthetic eucaryotes genomes encode multiple chaperonin genes which can be divided into α and β subtypes. Unlike the homo-oligomeric chaperonins from bacteria and mitochondria, chloroplast chaperonins are more complex and exists as intricate hetero-oligomers containing both subtypes. The Group I chaperonin requires proper interaction with a detachable lid-like co-chaperonin in the presence of ATP and Mg^2+^ for substrate encapsulation and conformational transition. Besides the typical Cpn10-like co-chaperonin, a unique co-chaperonin consisting of two tandem Cpn10-like domains joined head-to-tail exists in chloroplasts. Since chloroplasts were proposed as sensors to various environmental stresses, this diversified chloroplast chaperonin system has the potential to adapt to complex conditions by accommodating specific substrates or through regulation at both the transcriptional and post-translational levels. In this review, we discuss recent progress on the unique structure and function of the chloroplast chaperonin system based on model organisms *Chlamydomonas reinhardtii* and *Arabidopsis thaliana*. Knowledge of the chloroplast chaperonin system may ultimately lead to successful reconstitution of eukaryotic Rubisco *in vitro*.

## Introduction

Proteins are involved in almost all cellular processes. To attain biologically active functionality, newly-translated proteins must fold into a well-defined three-dimensional structure with high efficiency and fidelity. How proteins find folding trajectory to reach their native conformation is a fundamental question (Bartlett and Radford, 2009; Dill and MacCallum, 2012). Anfinsen's exquisite ribonuclease A renaturation assay reveals that the physical driving force of protein folding is encoded in its amino acid sequence, which suggests newly translated proteins are able to fold spontaneously *in vitro* (Anfinsen et al., 1954). However, proteins may expose unburied hydrophobic regions to a highly crowded environment during synthesis and folding, resulting in susceptibility to nonnative interaction that ultimately leads to misfolding and aggregation. Moreover, cells often encounter stresses such as high temperature, reactive oxygen species, and osmotic pressure, which may trap newly translated proteins in partially folded and aggregation-prone intermediates, or even terminally misfolded states (Ellis and Minton, 2006; Powers et al., 2009).

To counteract these stresses, cells have evolved a network of molecular chaperones as part of the protein homeostasis system to assist in protein *de novo* folding and maintain mature proteins in their native conformation (Hartl and Hayer-Hartl, 2002; Bukau et al., 2006; Hartl et al., 2011; Kim et al., 2013b; Saibil, 2013). The definition of molecular chaperone covers a wide range of proteins, including those accompanying proteins during synthesis and translocation, helping proteins cope with stress-induced misfolding and aggregation, or assisting protein complex assembly without being retained as part of the final structure of the protein. Chaperones also play an initiating role in protein unfolding and disaggregation or targeting misfolded proteins for degradation. Several families of ATP-dependent molecular chaperones exist in cells, with many of them classified as heat shock proteins (Hsps) since their expression is induced under conditions of high temperature. These chaperones are classified into four basic groups according to their molecular weight: Hsp60, Hsp70, Hsp90, and Hsp100. In addition to well-studied ATP-dependent molecular chaperones, a number of chaperones that assist in protein folding independent of ATP hydrolysis have also been identified (Suss and Reichmann, 2015; Horowitz et al., 2017). The entire cellular chaperone network composed of various molecular chaperones functions in diverse aspects of the protein quality control system to maintain protein homeostasis.

Chaperonins are one of the most important molecular chaperones that can be found in both prokaryotes and eukaryotes (Yébenes et al., 2011). They are large oligomeric protein complexes comprised of two rings stacked back to back, each of which creates a central cavity, known as the Anfinsen cage, for encapsulating substrate proteins. Two distantly related subgroups of chaperonins can be distinguished based on structure and functional dependence on co-chaperonin. Group I chaperonins, also known as Hsp60s, are present in bacteria and endosymbiotic organelles of eukaryotes: chloroplasts and mitochondria. They functionally cooperate in an ATP dependent manner with Hsp10 family proteins, which form the lid of the protein folding cage. This cooperation between Hsp60 and Hsp10 prevents substrate proteins from escaping and expands the folding chamber to accommodate larger proteins (Thirumalai and Lorimer, 2001; Horwich, 2013). Group II chaperonins, known as thermosome and TRiC, are found in archaea and the eukaryotic cytosol respectively. In contrast to Group I chaperonins, they contain a built-in lid instead of an obligate co-chaperonin that closes the folding chamber upon ATP binding. Accumulative studies of structure and function of Group II chaperonins from *Thermoplasma acidophilum, Saccharomyces cerevisiae*, and *Homo sapiens* revealed how exactly these protein machines work (Horwich et al., 2007; Lopez et al., 2015).

Knowledge about the functional mechanism of Group I chaperonins is mainly derived from the stable and simplified archetype GroEL/ES from *Escherichia coli* (Chan and Dill, 1996; Sigler et al., 1998; Hayer-Hartl et al., 2016). Compared to its counterpart in bacteria, the chloroplast chaperonin system is far more complicated due to its subunit diversification and dynamic nature (Hill and Hemmingsen, 2001; Weiss et al., 2009; Vitlin Gruber et al., 2013a; Trösch et al., 2015). Further investigation of the chloroplast chaperonin system will enhance our knowledge of chaperonins and may provide clues to remold this protein folding machine for specific purposes in synthetic biology.

## Group I chaperonin paradigm GroEL-GroES

GroEL and its cofactor GroES from *Escherichia coli* are the archetype of Group I chaperonin protein folding machines. Detailed structures of GroEL/GroES have been well studied over the last two decades by X-ray crystallography and cryo-electron microscopy. Like all Group I chaperonins, GroEL is a cylindrical tetradecamer composed of two heptameric rings which contain seven identical ~57 kD subunits. Each subunit is folded into three distinct domains: an equatorial domain haboring ATPase activity and providing almost all inter-ring and intra-ring contacts (Braig et al., 1994; Boisvert et al., 1996), an apical domain that binds co-chaperonin GroES and non-native substrate protein, and a hinge-like intermediate domain which connects the above two domains and is responsible for the allosteric signal transmission triggered by nucleotide binding and hydrolysis in the individual GroEL subunit (Xu et al., 1997; Ranson et al., 2006) (Sigler et al., 1998; Hayer-Hartl et al., 2016). The co-chaperonin, GroES, is a dome-shaped heptameric ring consisting of seven ~10 kD subunits (Hunt et al., 1996). Through its mobile loop region, GroES functionally interacts with helix H and helix I of the GroEL apical domain in the presence of nucleotide. The interaction between GroES and GroEL drives the conformational change of GroEL, mainly via twisting and elevating the apical domains, resulting in a two-fold increase in volume, which is sufficient to accomodate ~60 kDa protein substrates. The interaction also creates a protective hydrophilic cavity with a negatively-charged inner wall conducive to protein folding (Xu et al., 1997; Clare et al., 2012).

**Figure 1:**
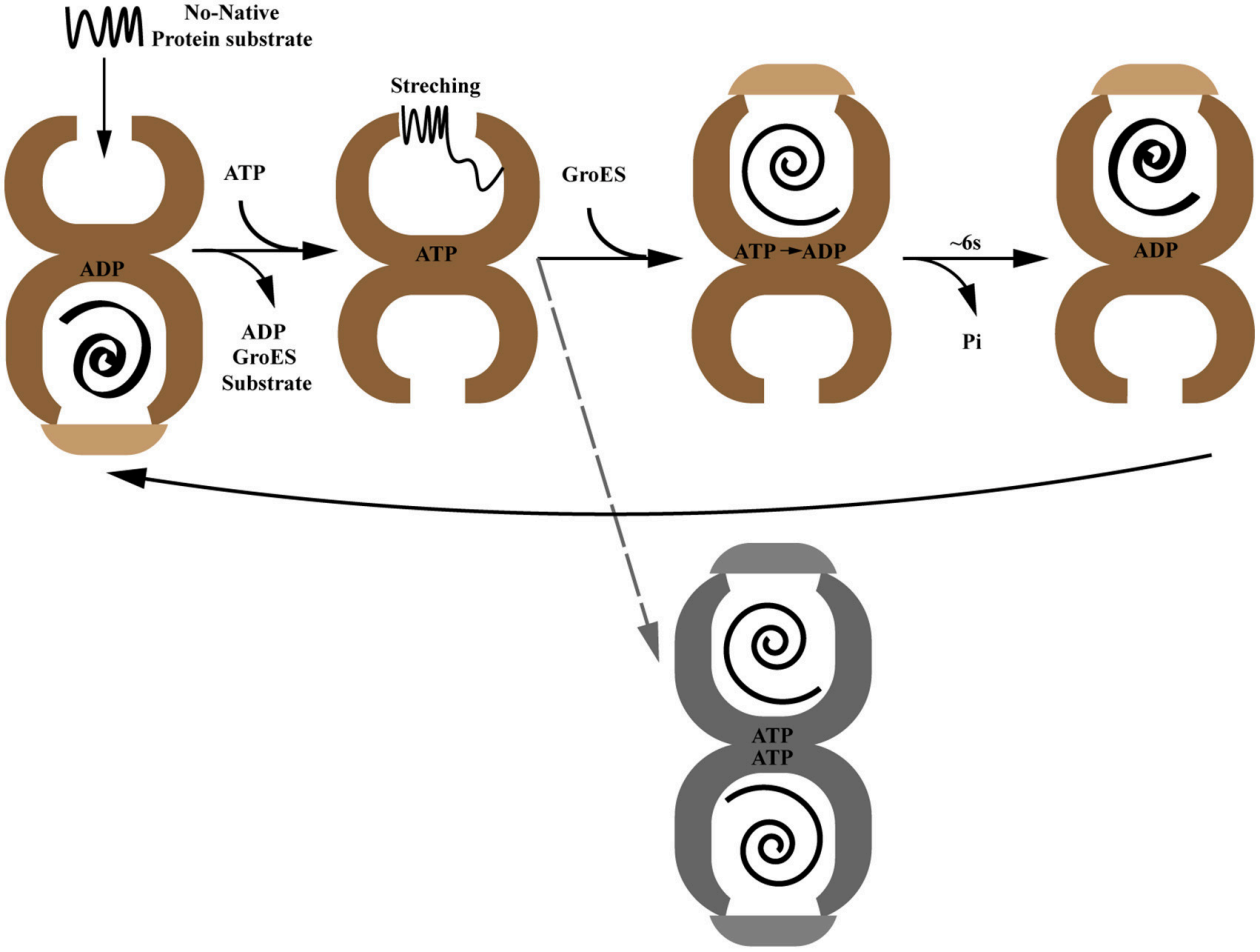
The paradigmatic chaperonin GroES/GroEL reaction cycle. The gray dotted line indicates the controversial GroEL/(GroES)2 folding active intermediate.

In addition to the static point-in-time structures of GroEL, the dynamic process of GroEL-GroES assisted protein folding has also been established by structural and biochemical studies. The protein folding reaction cycle driven by ATP binding and hydrolysis is governed by a precise cooperative network including inner-ring positive cooperativity and inter-ring negative cooperativity (Gray and Fersht, 1991; Bochkareva et al., 1992; Bochkareva and Girshovich, 1994) (Figure 1). In the apo-state, GroEL subunits switch back and forth between a tense T state (low affinity for ATP) and a relaxed R state (high affinity for ATP) (Ranson et al., 2006; Clare et al., 2012). In the protein folding state, the open nucleotide-free trans-ring captures non-native polypeptides with exposed hydrophobic surfaces. This interaction involves 3 or 4 GroEL apical domains which account for their overlap binding with both substrate protein and GroES. Followed by ATP binding, the substrate protein experiences a mechanical stretching by the conformational change of apical domains, which leads to unfolding of misfolded protein intermediates (Farr et al., 2000; Ashcroft et al., 2002; Horst et al., 2005; Elad et al., 2007; Lin et al., 2008). Then GroES collides with this ATP-occupied substrate-bound GroEL ring, called the cis-ring, forming a ternary structure. This triggers a large rigid body elevation and twist of apical domains that propels non-native polypeptide into a GroES-capped, hydrophilic chamber for folding (Chen et al., 2013). The process time of this step depends on the ATP hydrolysis rate, ~6 s at 25°C (Sharma et al., 2008). Subsequent binding of ATP in the opposite trans-ring results in GroES dissociation, as well as substrate protein and ADP release. At the same time, the opposite trans-ring becomes the new folding-active cis-ring (Ranson et al., 2006). For substrate proteins that are too large to be encapsulated (usually in excess of 60 kD), GroEL/GroES may still assist them in folding through binding and release from the trans-ring (Farr et al., 2003; Chaudhuri et al., 2009). Although the classical reaction cycle presented above depicts a perfect asymmetrical working model of the GroEL/GroES system, symmetrical football shaped GroEL/(GroES)_2_ complexes have also been observed in extensive studies suggesting the presence of GroEL with both chambers simultaneously active in folding substrates *in vivo* (Azem et al., 1994; Harris et al., 1994; Llorca et al., 1994; Sameshima et al., 2008, 2010). Recent crystal structures of symmetrical GroEL/(GroES)_2_ and mitochondrial Hsp60-(Hsp10)_2_ indicate this may be a conserved mechanism (Fei et al., 2014; Koike-Takeshita et al., 2014; Nisemblat et al., 2015). However, a fluorescence cross-correlation study showed that the GroEL/(GroES)_2_ structure is not likely to exist in the presence of physiological levels of ATP which leaves this mechanism still under debate (Haldar et al., 2015).

Despite the study of the GroEL/ES paradigm providing an insightful perspective on how Group I chaperonin functions as a protein folding machine, several key problems remain elusive. What structural features of a protein determine whether or not it is GroEL-dependent? How can GroEL balance its capability between specialization and generalization? What is the co-evolutionary process of Group I chaperonin and protein substrates? The study of organelle chaperonin systems may give us hints toward answering these questions.

## Chloroplast chaperonin and co-chaperonin proteins

Dating back to the 1980s when John Ellis at the University of Warwick studied light-driven protein synthesis in isolated intact chloroplasts, he observed the unexpected phenomenon that radioactive Rubisco large subunit (RbcL) co-migrates with another prominently stained band of protein before it interacts with transmembrane imported Rubisco small subunit (RbcS) to form Rubisco holoenzyme (Barraclough and Ellis, 1980). This Rubisco large subunit-binding protein was the first identified protein that binds to newly-synthesized polypeptides and subsequently became widely known as chaperonin Cpn60 (Hemmingsen and Ellis, 1986; Hemmingsen et al., 1988). Now we know that newly translated Rubisco large subunit was captured by chaperonin to prevent aggregation as a transient intermediate. Despite Cpn60 important role in folding Rubisco, its counterpart from *E. coli*, the GroEL/ES system, with the advantages of high stability and simple components eventually became a research paradigm that established the current model of the mechanism of chaperonin function as described above.

**Figure 2:**
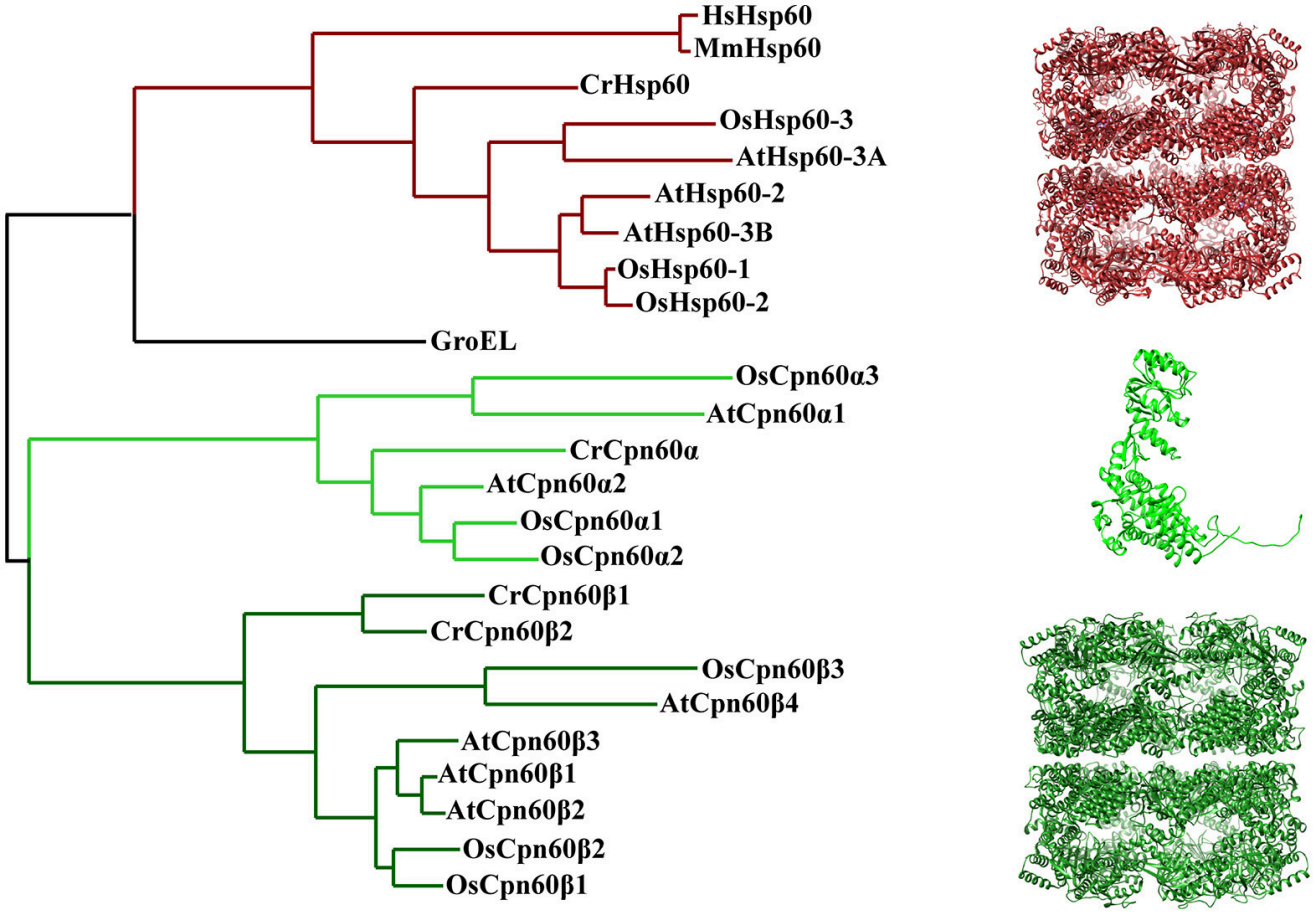
Phylogenetic relationships of chaperonin proteins from bacteria, chloroplasts, and mitochondria. The tree was generated using Phylogeny (http://www.phylogeny.fr/). Protein sequences of *E. coli, H. sapiens*, and *M. musculus* chaperonin are from UniProt database [GroEL (P0A6F5), HsHsp60 (P108090), MmHsp60 (P63038)]. Protein sequence of plant mitochondria and chloroplast chaperonins are from Phytozome, TAIR and RGAP [CrHsp60 (Cre06.g309100), AtHsp60-2 (AT2G33210), AtHsp60-3A (AT3G13860), AtHsp60-3B (AT3G23990), OsHsp60-1 (Os10g32550), OsHsp60-2 (Os03g04970), OsHsp60-3 (Os05g46290), CrCpn60α (Cre04.g231222), CrCpn60β1(Cre17.g741450), CrCpn60β2 (Cre07.g339150), AtCpn60α1 (At5g18820), AtCpn60α2 (At2g28000), AtCpn60β1 (At5g56500), AtCpn60β2 (At3g13470), AtCpn60β3 (At1g55490), AtCpn60β4 (At1g26230), OsCpn60α1 (Os12g17910), OsCpn60α2 (Os03g64210), OsCpn60α3 (Os09g38980), OsCpn60β1 (Os06g02380), OsCpn60β2 (Os02g01280), OsCpn60β3 (ChrSy.fgenesh.gene.28)]. The molecular structure was generated by UCSF Chimera (Pettersen et al., 2004) using CrCpn60β1coodinates 5CDI from Protein Data Bank.

Since chloroplast chaperonin subunits share ~50% sequence similarity with GroEL, it is reasonable to assume the functional mechanism of chloroplast chaperonin assisted protein folding is parallel to that of GroEL-ES mediated folding in bacteria. However, chloroplast chaperonins possess a unique feature that is not shared with chaperonins from bacteria and mitochondria; namely, multiple copies of two chaperonin subunit subtypes, α type and β type, which share ~50% sequence similarity with each other, are combined into hetero-oligomeric species (Musgrove et al., 1987). For example, the unicellular green algae *Chlamydomonas reinhardtii* encodes three CPN60 subunits, termed CPN60α1, CPN60β2, and CPN60β2 (Thompson et al., 1995; Schroda, 2004). Furthermore, the situation becomes even more complex in higher plants, such as monocotyledon and dicotyledon model organisms *Oryza sativa* and *Arabidopsis thaliana*, which both have six Cpn60 paralogs (Figure 2; Table 1) (*Arabidopsis* Cpn60 nomenclature in this review is according to the TAIR database) (Hill and Hemmingsen, 2001; Kim et al., 2013a). The recombinantly-expressed Cpn60β subunit from *Brassica napus* is able to assemble efficiently into a tetradecamer and fold the cyanobacterial Rubisco large subunit in *E. coli* cells, while the Cpn60α subunit is only capable of assembling into an oligomeric state and supporting folding in the presence of Cpn60β (Cloney et al., 1992a,b). An *in vitro* assay of Cpn60β1, Cpn60β2, Cpn60β3 from *Arabidopsis thaliana* (note: the protein nomenclature is in accordance with TAIR) showed that all three Cpn60β subunits assembled into β-type homo-oligomers and displayed refolding activity when cooperating with authentic chloroplast co-chaperonins (Vitlin et al., 2011). Each homo-oligomeric Cpn60β complex has its specific properties and preferences for co-chaperonin partners. Similarly, CPN60β2 and CPN60β1 from *Chlamydomonas* could be reconstituted into homo-oligomeric species *in vitro*, however, only CPN60β2 disassembled into monomer upon ATP hydrolysis (Bai et al., 2015). These results suggested that the Cpn60β subunits from one organism are functionally diverse though they share very high homology. Chloroplast chaperonins isolated from different organisms suggested they are α/β mixed hetero-oligomers, even though the arrangement of different subunits in the Cpn60 complex remains elusive (Cook et al., 1987; Musgrove et al., 1987; Hernan and Sligar, 1995; Nishio et al., 1999; Bai et al., 2015). *In vitro* reconstitution experiments with *E. coli* expressing Cpn60α and Cpn60β subunits from *Pisum sativum* generated two kinds of tetradecamers, α/β mixed hetero-oligomers and β homo-oligomers. Despite β subunits being able to assemble into homo-oligomers, they are preferentially incorporated into α/β mixed hetero-oligomers in the presence of α subunits. This provided strong support for the viewpoint that α/β mixed hetero-oligomers are predominant *in vivo* (Dickson et al., 2000). A recent study of CPN60 from *Chlamydomonas reinhardtii* also suggested that even though CPN60 monomers and homo-oligomers both possessed ATPase activity, only protein complexes containing all three subunits, the CPN60αβ1β2 oligomeric complex, have functional cooperation with GroES in refolding a model substrate (Bai et al., 2015). Thus, overwhelming evidence suggests that the major functional species *in vitro* is a hetero-oligomer composed of α and β subunits.

**Table 1:** Chloroplast chaperonins and co-chaperonins in model species: nomenclature and function.

**Protein name**	**Organism**	**Gene number**	**Mutant line**	**Phenotype**	**References**
CrCpn60α	*Chlamydomonas reinhardtii*	Cre04.g231222	Unknown	Unknown	
CrCpn60β1	*Chlamydomonas reinhardtii*	Cre17.g741450	Unknown	Unknown	
CrCpn60β2	*Chlamydomonas reinhardtii*	Cre07.g339150	Unknown	Unknown	
AtCpn60α1	*Arabidopsis thaliana*	At2g28000	T-DNA insertion (*slp*1)	Retardation of embryo development before the heart stage	Apuya et al., 2001
AtCpn60α2	*Arabidopsis thaliana*	At5g18820	T-DNA insertion (*emb3007*)	Embryo development arrested at the globular stage	Ke et al., 2017
AtCpn60β1	*Arabidopsis thaliana*	At1g55490	T-DNA insertion (*len1*)	Impaired leaves and showed systemic acquired resistance (SAR) under short-day condition	Ishikawa et al., 2003
AtCpn60β2	*Arabidopsis thaliana*	At3g13470	T-DNA insertion	No obvious phenotype	Suzuki et al., 2009
AtCpn60β3	*Arabidopsis thaliana*	At5g56500	Unknown	Unknown	
AtCpn60β4	*Arabidopsis thaliana*	At1g26230	Ds transposon-tagged lines(*crr27)*	Defective in NDH activity	Peng et al., 2011
OsCpn60α1	*Oryza Sativa*	Os12g17910	T-DNA insertion	Pale-green phenotype at the seedling stage	Kim et al., 2013a
OsCpn60α2	*Oryza Sativa*	Os03g64210	Natural mutation	Albino phenotype before the 3-leaf stage grown below 24°C	Jiang et al., 2014
OsCpn60α3	*Oryza Sativa*	Os09g38980	Unknown	Unknown	
OsCpn60β1	*Oryza Sativa*	Os06g02380	Unknown	Unknown	
OsCpn60β2	*Oryza Sativa*	Os02g01280	Unknown	Unknown	
OsCpn60β3	*Oryza Sativa*	ChrSy.fgenesh.gene.28	Unknown	Unknown	
CrCpn11	*Chlamydomonas reinhardtii*	Cre16.g673729	Unknown	Unknown	
CrCpn20	*Chlamydomonas reinhardtii*	Cre08.g358562	Unknown	Unknown	
CrCpn23	*Chlamydomonas reinhardtii*	Cre12.g505850	Unknown	Unknown	
AtCpn10-1	*Arabidopsis thaliana*	At3g60210	Unknown	Unknown	
AtCpn10-2	*Arabidopsis thaliana*	At2g44650	Unknown	Unknown	
AtCpn20	*Arabidopsis thaliana*	At5g20720	T-DNA insertion	Increased ABA sensitivity, homozygous lethal	Zhang et al., 2013
OsCpn10	*Oryza Sativa*	Os10g41710	Unknown	Unknown	
OsCpn20-1	*Oryza Sativa*	Os02g54060	Unknown	Unknown	
OsCpn20-2	*Oryza Sativa*	Os09g26730	Unknown	Unknown	
OsCpn20-3	*Oryza Sativa*	Os06g09679	Unknown	Unknown	
OsCpn20-4	*Oryza Sativa*	Os06g09688	Unknown	Unknown	

*Chloroplast chaperonin names from Chlamydomonas reinhardtii, Arabidopsis thaliana, are according to Phytozome, TAIR, while those from Oryza Sativa are based on the original names described in Kim et al. (2013a). Chloroplast co-chaperonin names from Chlamydomonas reinhardtii, Arabidopsis thaliana are based on the original names described in Tsai et al. (2012) and Vitlin Gruber et al. (2013a). Chloroplast co-chaperonins from Oryza Sativa are named in this review*.

Another feature that is unique to hetero-oligomeric chloroplast chaperonins is their notorious instability in the presence of ATP, that is, the purified Cpn60 complex from *Pisum sativum* and recombinantly expressed CPN60αβ1β2 of *Chlamydomonas reinhardtii* display ATP-dependent dissociation (Dickson et al., 2000; Bai et al., 2015). The oligomer dissociation largely results from the interaction of equatorial domains.

**Figure 3:**
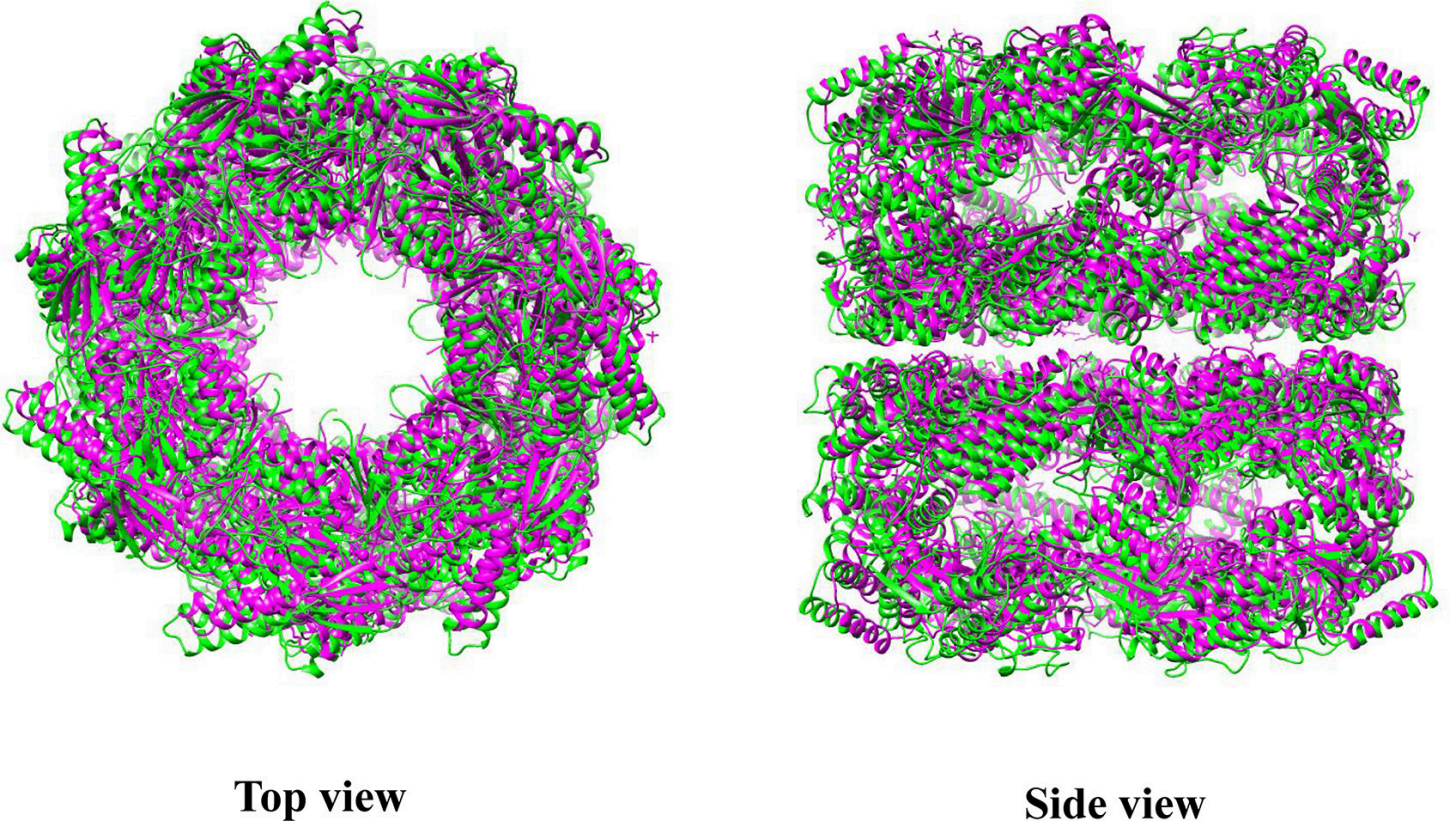
Superposition of CrCpn60β1 with GroEL. Green represents CrCpn60β1 and magenta represents GroEL. The molecular structure was generated by UCSF Chimera (Pettersen et al., 2004) using CrCpn60β1 coordinates 5CDI and GroEL coordinates 1XCK from Protein Data Bank.

Electron micrographs of the Cpn60αβ hetero-oligomer reveal that chloroplast chaperonin exhibits the well-known double-ring cylindrical shape, indicating a conserved structure in the Group I chaperonin kingdom (Dickson et al., 2000). Recently, the first crystal structure of the homo-oligomer CPN60β1, which shows partial functionality in the presence of Hsp10, was solved at 3.8 Å. The overall architecture of CPN60β1 displays a typical type I chaperonin structure, with a 7-fold symmetrical cylinder structure consisting of two stacked rings composed of seven subunits. Each subunit is also composed of an equatorial, intermediate, and apical domain. In Cpn60 subunits, the equatorial domain directs oligomer formation and the C-terminus (484-547) in this domain determines oligomer disassembly properties driven by ATP hydrolysis (Bai et al., 2015; Zhang et al., 2016a). However, Apo CPN60β1 resembles the intermediate state of allosteric GroEL, with a central cavity 6 Å larger than GroEL in diameter (Zhang et al., 2016a) (Figure 3). Moreover, the compaction in CPN60β1 is looser relative to GroEL, with less inter-subunit interface area and fewer amino acids involved in inter-subunit contacts. One distinguishing feature of CPN60β1 is that it has a wider ATP binding pocket compared to apo GroEL. These structural features may explain Cpn60 specific dissociation driven by ATP hydrolysis.

**Figure 4:**
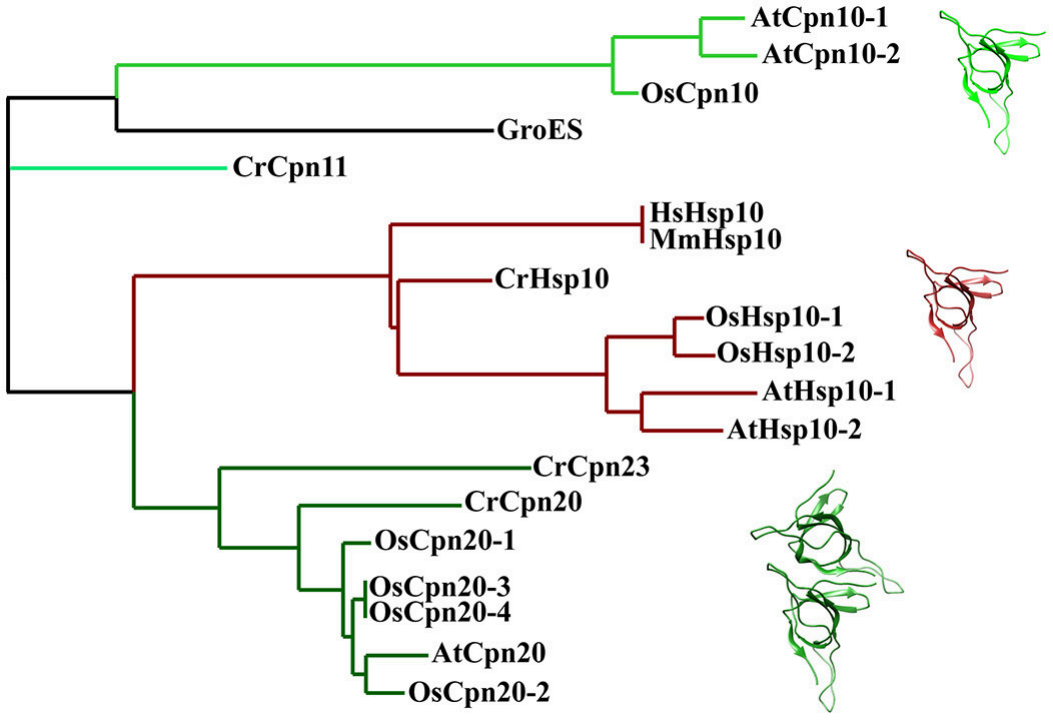
Phylogenetic relationships of co-chaperonin proteins from bacteria, chloroplasts and mitochondria. The tree was generated using Phylogeny (http://www.phylogeny.fr/). Protein sequences of *E. coli, H. sapiens*, and *M. musculus* chaperonin are from UniProt database Protein sequence of plant mitochondria and chloroplast co-chaperonins are from Phytozome, TA Protein sequences of mitochondria chaperonin are from IR and RGAP [AtHsp10-1 (AT1G14980), AtHsp10-2 (AT1G23100), CrHsp10 (Cre03.g178450), OsHsp10-1(Os03g25050), OsHsp10-2 (Os07g44740), CrCpn11 (Cre16.g673729), CrCpn20 (Cre08.g358562), CrCpn23 (Cre12.g505850), AtCpn10-1 (At3g60210), AtCpn10-2 (At2g44650), AtCpn20 (At5g20720), OsCpn10 (Os10g41710), OsCpn20-1 (Os02g54060), OsCpn20-2 (Os09g26730), OsCpn20-3 (Os06g09679), OsCpn20-4 (Os06g09688)]. The molecular structure was generated by UCSF Chimera (Pettersen et al., 2004) using GroES coordinates 1AON from Protein Data Bank.

Compared to their bacterial and mitochondrial homologs, chloroplast co-chaperonin subunits also exhibit interesting difference. In 1992, the first chloroplast co-chaperonin was identified by a pull-down assay using pea chloroplast lysate with GroEL as bait. This chloroplast co-chaperonin is capable of assisting GroEL in folding a chemically denatured dimeric Rubisco, similar to GroES. But a fascinating aspect of this chloroplast co-chaperonin is that its molecular weight is ~24 kD, twice the size of GroES (Bertsch et al., 1992). Similarly, co-chaperonin AtCpn21 with a molecular weight of ~21 kD has also been observed in chloroplasts of *Arabidopsis thaliana*. The AtCpn21 precursor protein deduced by cDNA sequence contains a typical chloroplast transit peptide at its amino-terminus and two GroES-like domains joined together head-to-tail (Hirohashi et al., 1999). Mature AtCpn21 protein formed tetrameric structures as revealed by gel-filtration and cross-linking analysis (Koumoto et al., 1999). In addition to Cpn20s, classical GroES-like co-chaperonins have also been found in chloroplasts of several organisms (Schlicher and Soll, 1996; Hill and Hemmingsen, 2001). Since whole genome information of multiple plant species is available, it is known that there are two types of co-chaperonin subunits present in chloroplasts: a conventional GroES-like Cpn10 type, and a chloroplast-specific Cpn20 type that contains two tandem GroES-like domains (Figure 4). *Chlamydomonas reinhardtii* encodes three chloroplast co-chaperonin subunits named according to their molecular weights as CrCPN11, CrCPN20, and CrCPN23, while *Arabidopsis thaliana* and *Oryza sativa* both have three paralogs as listed in Table 1. Though chloroplast co-chaperonin subunits seem conserved among species, each subunit has unique biochemical properties. In *Arabidopsis*, AtCpn10-2 and AtCpn20 form functional homo-oligomers on their own, while AtCpn10-1 subunit is functional only upon formation of hetero-oligomers with other co-chaperonins (Vitlin Gruber et al., 2014). The case is similar in *Chlamydomonas*, that is, CrCPN20 and/or CrCPN23 tend to combine with CrCPN10 to form functional hetero-oligomers composed of seven GroES-like domains (Tsai et al., 2012).

Although some mitochondria and bacteria also possess more than one chaperonin subunit, it is still a fascinating question why the chloroplast uniquely contains divergent Cpn60 α/β chaperonin subunits as well as Cpn10/20 co-chaperonin types (Kumar et al., 2015). Transcriptome and proteome studies in *Arabidopisis* indicated expression levels of chaperonin and co-chaperonin genes differ according to developmental stage (Weiss et al., 2009), which increases the complex potential of the chaperonin system for regulation on both transcriptional and post-translational levels. In different tissues or developmental stages, or even facing different environmental stimuli, it is plausible that the functional chaperonin system is composed of various combinations of chloroplast chaperonin and its co-chaperonins to strategically deal with specific situations. From an evolutionary perspective, the chloroplast is an endosymbiotic organelle where the most important chemical reaction of photosynthesis takes place. It is also reasonable to deduce that chloroplasts developed a chaperonin system with several features that adapt to accommodate different photosynthetic proteins. Our knowledge from the Group I chaperonin paradigm, the GroEL-ES system, is insufficient to explain the multiformity of the chloroplast chaperonin system. Therefore, genetic, biochemical and structural data directly obtained from chloroplast chaperonins will be needed to shed light on the mechanism of this protein folding machine in photosynthesis.

## Functional divergence of chloroplast chaperonin and co-chaperonin subunits

Chloroplast chaperonins are extremely labile protein complexes, and therefore conventional biochemical methods may fall short to when it comes to explaining the nature of their multiplicity. Genetic analysis of chloroplast chaperonin and co-chaperonin mutants and the study of their roles in specific tissues or developmental stages provide a global view on how this dynamic chaperonin system works and the possible significance of its divergence. The first phenotypic dissection of Cpn60 mutants was conducted in *cpn60α1* (At2g28000) which was generated by T-DNA insertion in *Arabidopsis*. This *atcpn60α1* mutant was termed *schlepperless* due to its highly reduced embryonic cotyledons. Compared to wild-type, the entire embryo of *Atcpn60α1* remains white during maturation, suggesting photosynthesis incompetence. Further analysis of this mutant indicates that the absence of functional AtCpn60α1 disrupts the development of the chloroplast which results in defective development of the embryo (Apuya et al., 2001). A similar function of Cpn60α has also been demonstrated in rice according to a study using forward genetics. Map based cloning of the thermo-sensitive chloroplast development 9 (tcd9) rice mutant revealed that the mutation is located in a gene encoding a Cpn60α protein. Genetic complementation demonstrated that the *OsCpn60*α gene is precisely responsible for the albino phenotype before the 3-leaf stage grown below 20°C (Jiang et al., 2014). These two studies suggest a conserved function of Cpn60α members in chloroplast development.

Additional Cpn60 mutants including *atcpn60α1* (At2g28000), *atcpn60β1* (At1g55490), and *atcpn60β2* (At3g13470) have been isolated in research focusing on chloroplast division in *Arabidopsis* (Suzuki et al., 2009). This work showed that AtCpn60α1, AtCpn60β1, and AtCpn60β2 are required for formation of a normal chloroplast division apparatus, especially by influencing folding of chloroplast division related proteins and regulating FtsZ polymer dynamics. The *atcpn60β1atcpn60β2* double mutant exhibited an albino phenotype, similar to the *atcpn60α1* single mutant. However, *atcpn60β1* and *atcpn60β2* single mutants did not show an albino phenotype but had slightly reduced chlorophyll. These results suggest that the phylogenetically closely related AtCpn60β1 and AtCpn60β2 are functionally redundant. Another notable observation is that although the *atcpn60α1* single mutant and *atcpn60β1atcpn60β2* showed a similar albino phenotype, *atcpn60β1atcpn60β2* was able to germinate while *cpn60α1* arrested at the embryo stage (Apuya et al., 2001; Suzuki et al., 2009). This supports the hypothesis that different Cpn60 subunits may incorporate into one major pathway; However, these subunits may also be individually responsible for folding specific protein substrates.

The hypothesis raised above was supported by a study identifying Rubisco activase interacting proteins during heat stress. A 60 kD protein with a N-terminal signal sequence simultaneously corresponding to both AtCpn60β1 and AtCpn60β2 was captured. Cpn60β was associated with Rubisco activase in a high molecular mass complex, and the dynamic regulation of their association depended on heat stress. This suggested AtCpn60β1 and/or AtCpn60β2 play a role in preventing Rubisco activase from thermal denaturation. The study of *Cpn60α1* mutant from *Oryza sativa* provided another example; the amount of Rubisco large subunit (rbcL) was severely reduced in the *osCpn60α1* mutant, while some imported proteins remained unchanged. This demonstrated that Rubisco large subunit may depend on OsCpn60α1 for proper folding (Kim et al., 2013a). The direct evidence for the assumption came from the study of Cpn60β4 in *Arabidopsis*. When the *Cpn60β4* (At1g26230) gene is defective, the chloroplast fails to accumulate the NADH dehydrogenase-like complex (NDH), and the other three Cpn60β subunits cannot replace the function of Cpn60β4. Co-immunoprecipitation data revealed that Cpn60β4 forms a hetero-oligomeric complex with other Cpn60 α and β subunits and this complex is essential for the folding of the NDH subunit NdhH. Furthermore, the unique C-terminus of Cpn60β4 is required for the refolding activity of NdhH in the chaperonin complex (Peng et al., 2011). A very recent study about the function of Cpn60α2 (At5g18820) during *Arabidopsis* embryo development provided another example of subunit specific folding of protein substrates. A co-immunoprecipitation assay coupled with LC-MS/MS identified KASI, a protein important for the formation of heart-shaped embryos, as a specific interactor of Cpn60α2. A genetic study showed that KASI protein levels were largely reduced in the *atcpn60α2* mutant. Further studies demonstrated that Cpn60α2, Cpn60β2, and Cpn60β3 were able to assemble into a functional chaperonin complex and specifically assist in folding of KASI. It is plausible that these three subunits form functional oligomers in certain developmental stages. However, a detailed biochemical characterization remains elusive (Ke et al., 2017).

A biochemical study of chloroplast chaperonins from *Chlamydomonas reinhardtii* provided additional insight into the divergence of CPN60 subunits. Specifically, domain swapping between GroEL and CPN60 subunits demonstrated that in the functional hetero-oligomeric complex CPN60αβ1β2, the CPN60α apical domain could not functionally cooperate with co-chaperonin GroES, but recognized its cognate substrate CrRubisco large subunit more efficiently than CPN60β apical domain and vice versa. This implied chloroplast chaperonin adopts a different strategy than GroEL to cope with the paradox that the same region of apical domains is responsible for simultaneous binding of co-chaperonin and protein substrates (Chen et al., 2013; Zhang et al., 2016b). Though functionality of two types of subunits is divergent, they are highly cooperative in oligomer formation. The equatorial domain of CPN60α could not direct self-assembly, but cooperated with CPN60β1 to form fully functional oligomers (Zhang et al., 2016a).

It has long been accepted that specific co-chaperonins would endow the chaperonin system with uncommon ability to accommodate diverse protein substrates. An interesting example is the T4 phage encoded protein, GP31, which is homologous to GroES. Structural and biochemical studies of GP31 proved that an expanded folding chamber was formed with GroEL-GP31 and these heterologous partners are able to fold the capsid protein GP23. Similar mechanisms may exist in chloroplast considering that there are two kinds of co-chaperonin isoforms (Figure 4). A study characterizing chloroplast co-chaperonin subunits of both *Arabidopsis* and *Chlamydomonas* indicated that different combinations of Cpn10/20 subunits create diverse hetero-oligomers with various refolding activities, perhaps adapting the chaperonin system to specific protein substrates (Tsai et al., 2012).

**Figure 5:**
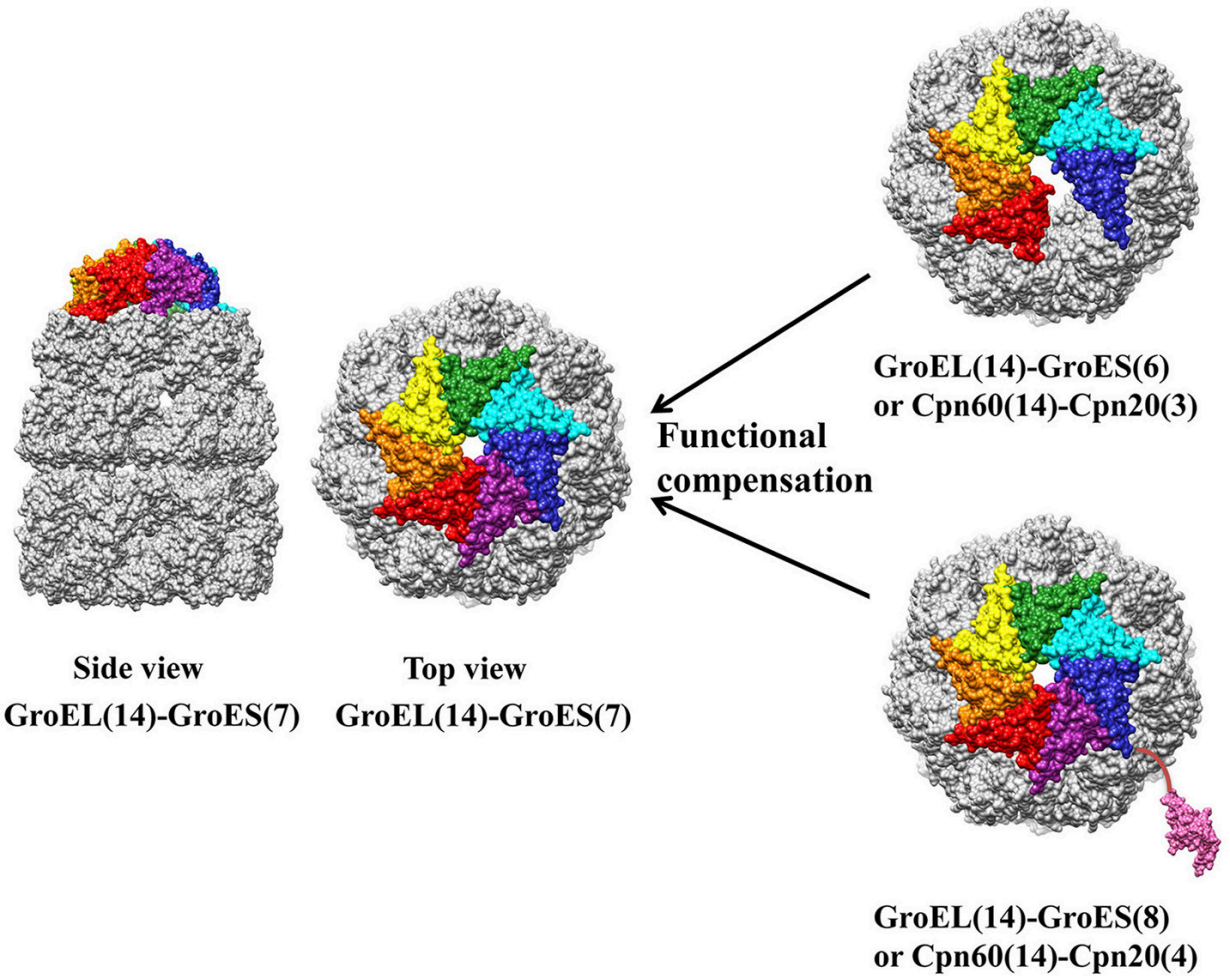
Asymmetric functional interaction between chloroplast chaperonin and co-chaperonin. GroEL is colored in gold, and seven GroES subunits are colored in red, orange, yellow, green, cyan, blue, and pink. Co-chaperonins consisting of six or eight GroES like domains function equally as well as heptameric GroES. The molecular structure was generated by UCSF Chimera (Pettersen et al., 2004) using GroES-GroEL-ADP coordinates 1AON from Protein Data Bank.

Study of chloroplast co-chaperonin gene mutants in *Arabidopsis* addressed the unique importance of Cpn20 type co-chaperonin. Cpn10 type co-chaperonin null mutants such as *atcpn10-1* and *atcpn10-2* were able to germinate normally, whereas knock out of Cpn20 in Arabidopsis is lethal (Zhang et al., 2013). It has been demonstrated that Cpn20 homo-oligomer is able to cooperate with chaperonin, which also raises the symmetry dilemma. Namely, how does hexameric or octameric Cpn20 oligomer interact with a chaperonin complex with seven-fold symmetry? Two studies suggested this is a simple obstacle that the chaperonin system overcomes. Cpn20 from *Plasmodium falciparum* apicoplast, a degenerate chloroplast, is fully functional *in vitro* and able to replace GroES in *E. coli* at both normal and heat-shock temperatures. Since *Plasmodium falciparum* apicoplast contains only one Cpn20 type co-chaperonin, PfCpn20 may also function similarly *in vivo* (Vitlin Gruber et al., 2013b). In another *in vitro* biochemical study, GroES and Cpn20 concatamers, consisting of six to eight covalently linked 10 kD GroES domains, cooperatively function with GroEL similar to the native heptameric GroES form. The cooperation between chaperonin and co-chaperonin results from asymmetrical interaction by leaving one chaperonin subunit unbounded (six GroES domain) or excluding one co-chaperonin from the interaction (eight GroES domain) (Figure 5) (Guo et al., 2015). These results showed how chloroplast Cpn20, with even-numbered GroES-like domains, cooperated with odd-numbered chaperonin oligomers. However, though concatamers composed of six or eight GroES domains are functional, it seems that the native form of co-chaperonin in *Arabidopsis* and *Chlamydomonas* is most likely a hetero-oligomer with seven-fold symmetry (Tsai et al., 2012; Vitlin Gruber et al., 2014).

Another deduction on why chloroplasts have so many chaperonin and co-chaperonin genes points to their additional functions other than folding proteins as molecular chaperones, so-called moonlighting function. For example, CPN60α was previously reported to exhibit a novel function as a group II intron-specific binding protein and was presumed to be a general chloroplast RNA splicing factor (Balczun et al., 2006). Chloroplast proteomic studies in *Arabidopsis* showed that Cpn20 is much more abundant than other subunits in the chloroplast chaperonin system, which suggested Cpn20 has additional moonlighting function (Peltier et al., 2006). Cpn20 overexpressing mutants and mutants with defective co-chaperonin activity were reported to increase FeSOD activity by functioning as probable Fe chaperones (Kuo et al., 2013a,b). Analysis of Cpn20 knock down mutants showed that Cpn20 functions negatively in the ABAR-WRKY40 coupled ABA signaling pathway by antagonizing Mg-chelatase H subunit to derepress the ABA-responsive WRKY40 transcription repressor (Zhang et al., 2013, 2014). To clarify the moonlighting function of different chloroplast chaperonin and co-chaperonin proteins, more studies are still needed.

## Chloroplast chaperonin assisted rubisco folding and assembly

Ribulose-1,5-bisphosphate carboxylase/oxygenase (Rubisco), the most important chloroplast chaperonin substrate, catalyzes the chemical reaction by which inorganic carbon enters the organic biosphere. As a key enzyme that catalyzes the rate-limiting step of photosynthetic carbon fixation in the Calvin–Benson–Bassham cycle as well as an ancient enzyme that evolved from a high CO_2_ atmospheric environment, Rubisco is widely known for its abundance and inefficiency. Therefore, numerous efforts have been undertaken to engineer Rubisco to improve carbon fixation efficiency with reduced amounts of Rubisco at the expense of less nitrogen. However, a high throughput Rubisco mutant screening platform is so far infeasible due to the lack of assembly of Form I Rubisco outside chloroplasts.

Form I Rubisco, a hexadecameric protein complex consisting of eight large (RbcL) and eight small (RbcS) subunits, exists in plants, green algae, cyanobacteria and proteobacteria. Even though it has been widely accepted that newly-translated RbcL would be captured by chloroplast chaperonin to avoid aggregation, how RbcS is coupled with RbcL and assembled into Rubisco holoenzyme had remained unknown. The breakthrough came from the study of RbcX protein which was first identified in *Anabaena* and *Synechococcus*. Co-expression of RbcX in *E. coli* facilitated the production of active Rubisco, suggesting RbcX is involved in the Rubisco assembly pathway (Li and Tabita, 1997; Onizuka et al., 2004; Emlyn-Jones et al., 2006). Functional analysis revealed that RbcX acts as an assembly chaperone, downstream of chaperonin-mediated RbcL folding, to promote the formation of RbcL(8) core complexes. The crystal structure showed that the 15 kD RbcX forms a homodimer containing a hydrophobic central groove that binds the peptide motif EIKFEFD present at the C-terminus of RbcL subunits. The subsequent cryo-electron microscopy structure of RbcL_(8)_-(RbcX_2_)_(8)_ assembly intermediate revealed RbcX_(2)_ acts as a molecular stapler in stabilizing the RbcL subunits and facilitates RbcL_(8)_ core assembly. Finally, replacement of RbcX by RbcS results in holoenzyme formation (Saschenbrecker et al., 2007; Liu et al., 2010). Highly homologous RbcX proteins exist in *Thermosynechococcus elongates* and *Arabidopsis thaliana* as revealed by their structures, implying this may be a conserved mechanism for Rubisco assembly across species (Tarnawski et al., 2011; Kolesinski et al., 2013).

In addition to RbcX, other Rubisco assembly associated factors have also been discovered in recent years. For example, analysis of *Zea mays* mutants showed that Bundle Sheath Defective 2 (Bsd2), Rubisco Accumulation Factor 1 (Raf1), and Rubisco Accumulation Factor 2 (Raf2) are responsible for proper assembly of Rubisco (Brutnell et al., 1999; Feiz et al., 2012, 2014). Co-transformation of Raf1 and RbcL from Arabidopsis into tobacco chloroplast results in improved production of hybrid Rubisco, suggesting Raf1 protein co-evolved with RbcL (Whitney et al., 2015). Just like RbcX, Raf1 also functions downstream of chaperonin-assisted RbcL folding by stabilizing RbcL dimers for assembly into (RbcL)_8_ core complexes, suggesting diverse Rubisco assembly factors have functional redundancy (Hauser et al., 2015).

Despite more and more Rubisco assembly factors being identified, recombinant production of plant Rubisco in *E. coli* or reconstitution of Rubisco holoenzyme in test tubes has not been achieved so far. From the perspective of evolution, it is noteworthy that α/β type divergence of chaperonin subunits appeared after the endosymbiotic event involving cyanobacteria with only GroEL-type chaperonin. Given the fact that Rubisco is the most abundant protein in the world, the chaperonin system responsible for Rubisco biogenesis must have specially adapted to cope with the burdensome task of folding and assembling such large quantities of protein. According to the classic Rubisco folding and assembly pathway described above, the chaperonin system is believed to function in RbcL folding, upstream of holoenzyme assembly, where GroEL could have done the job in prokaryotic organisms. However, the protein folding machine for plant Rubisco is the chloroplast chaperonin system, which we believe it has special properties cannot be replaced by GroEL system. Maybe it is time to set up an *in vitro* system containing the chloroplast chaperonin system and currently known Rubisco assembly factors to make plant Rubisco reconstitution a reality.

## Concluding remarks

Genetic and biochemical studies emphasize the regulatory role of chloroplast chaperonin in photosynthesis, and some photosynthetic proteins are identified as substrates of chloroplast chaperonin, such as Rubisco large subunit, NDH subunit NdhH and ATPase synthase γ subunits (Mao et al., 2015). The protein substrates involved in embryo development and chloroplast division, as well as the processes affected by Cpn60 subunit mutation are not yet clarified. Sophisticated studies are needed to identify the substrates specifically folded by certain chaperonin subunits. The crystal structure of CPN60β1 resembles that of GroEL (Zhang et al., 2016a), but the composition and arrangement of the *in vivo* chaperonin complex, which might vary under different conditions, remains elusive. Elucidating the functional mechanism of chloroplast chaperonin will be of special importance in the context of efforts to assemble eukaryotic Rubisco *in vitro*.

## Author contributions

All authors listed, have made substantial, direct and intellectual contribution to the work, and approved it for publication.

### Conflict of interest statement

The authors declare that the research was conducted in the absence of any commercial or financial relationships that could be construed as a potential conflict of interest. The reviewer CW and handling Editor declared their shared affiliation.
